# Favorable response to pembrolizumab after durvalumab failure in a stage III sarcomatoid carcinoma of the lung: a case report

**DOI:** 10.1186/s40360-020-00404-7

**Published:** 2020-04-03

**Authors:** Kazumi Nishino, Kei Kunimasa, Madoka Kimura, Takako Inoue, Motohiro Tamiya, Hanako Kuhara, Toru Kumagai

**Affiliations:** Department of Thoracic Oncology, Osaka International Cancer Institute, 3-1-69 Otemae Chuo-ku, Osaka, 541-8567 Japan

**Keywords:** Pulmonary sarcomatoid carcinoma, Durvalumab, Pembrolizumab, PD-L1, PD-L2

## Abstract

**Background:**

Pulmonary sarcomatoid carcinoma is a rare non-small-cell lung cancer (NSCLC) subtype with a poor prognosis. In the phase III PACIFIC study, durvalumab significantly improved progression-free survival and overall survival versus placebo, in patients with stage III NSCLC who do not have disease progression after concurrent chemoradiotherapy. However, treatments for patients who discontinue durvalumab due to disease progression, are unknown.

**Case presentation:**

We report a case of favorable response to pembrolizumab in a patient with disease progression during durvalumab consolidation therapy after chemoradiotherapy for stage III pulmonary sarcomatoid carcinoma with high programmed cell death ligand 1 (PD-L1) and PD-L2 expression.

**Conclusion:**

Here, we present what, to the best of our knowledge, is the first reported case in which durvarumab resistance after definitive chemoradiotherapy in a patient with stage III pulmonary sarcomatoid carcinoma was overcome by pembrolizumab.

## Background

Pulmonary sarcomatoid carcinoma (SC) is a rare subtype of non-small-cell lung cancer (NSCLC), accounting for approximately 0.1 to 0.4% of all lung cancer cases [1]. SC is a general term that includes pleomorphic carcinoma, spindle cell carcinoma, giant cell carcinoma, carcinosarcoma, and pulmonary blastoma [2]. SC shows highly aggressive biological behaviors associated with a poor prognosis and high resistance to chemotherapy [3, 4]. SC shows high levels of programmed death ligand-1 (PD-L1) [5, 6], and it has recently been reported that immune checkpoint inhibitors (ICIs) are very effective. Most ICIs are PD-1 inhibitors such as nivolumab and pembrolizumab [7]. In the phase III PACIFIC study, durvalumab significantly improved progression-free survival (PFS) and overall survival (OS) versus placebo, in patients with stage III without disease progression after concurrent chemoradiotherapy [8, 9]. Following discontinuation of durvalumab, 195 patients (41.0%) received subsequent anticancer therapy. Most patients subsequently received cytotoxic chemotherapy, and only 38 patients (8.0%) received additional immunotherapy [9]. No results have been reported for the subsequent treatment. We herein report the use of pembrolizumab in the setting of disease progression during durvalumab consolidation therapy after chemoradiotherapy in a patient with stage III SC with high PD-L1 expression.

## Case presentation

**Fig. 1:**
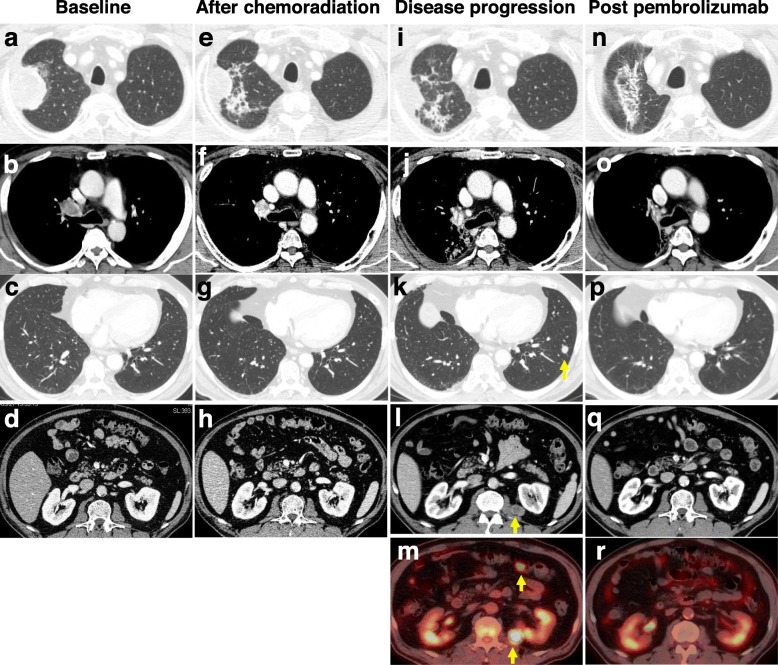
Chest computed tomography (CT) and 18F-fluorodeoxyglucose (FDG) positron emission tomography (PET)-CT. Imaging findings during the patient’s course (**a**, **b**, **c**, and **d**) at baseline (before chemotherapy), (**e**, **f**, **g**, and **h**) after chemoradiotherapy and before durvalumab consolidation therapy, (**i**, **j**, **k**, **l**, and **m**) after the seventh round of durvalumab, and (**n**, **o**, **p**, **q**, and **r**) after the ten cycles of pembrolizumab. **a** and **b** CT showing right upper lobe and hilum involvement at the time of diagnosis (May 2018). **e** and **f** CT showing the response to chemoradiotherapy (August 2018). **k** and **l** CT showing progressive disease during durvalumab therapy (November 2018). New metastatic nodules were visible in the left lower lobe (**k**, *arrow*) and the left psoas (**l**, *arrow*). **m** PET-CT showed FDG accumulation in the left psoas and the abdominal lymph node (*arrows*) (November 2018). **n**, **o**, **p**, **q**, and **r** Both primary and metastatic lesions were dramatically improved by pembrolizumab treatment

A 62-year-old healthy asymptomatic male current-smoker presented with an abnormal shadow on chest radiography during a regular health check-up. A computed tomography (CT) scan showed a mass in the right upper lobe. Transbronchial lung biopsy pathology confirmed SC. The lung biopsy specimens were negative for p40, thyroid transcription factor 1, and calretinin, and positive for cytokeratin AE1/3. The patient was diagnosed with stage IIIA (cT3N1M0) SC in May 2018. Molecular testing revealed no targetable mutations. Immunohistochemical staining of the tumor tissue showed PD-L1 expression in 90% of the tumor. The patient was treated with two cycles of concurrent vinorelbine (20 mg/m^2^ on days 1 and 8) plus cisplatin (`5 mg/m^2^ on day 1) and definitive 60 Gy of thoracic radiation therapy. He showed a partial response to treatment at the primary tumor site and received durvalumab at 10 mg/kg every 2 weeks. Three months later, in November 2018, disease progression was detected by 18F-fluorodeoxyglucose-positron emission tomography, which showed new metastases in the left lung, abdominal lymph nodes, and left psoas. He had undergone seven cycles of durvalumab. He immediately received pembrolizumab at 200 mg/body every 3 weeks, because of the high expression of PD-L1 in the tumors. After two cycles of pembrolizumab, CT revealed a durable clinical response in December 2018. The patient has subsequently achieved complete tumor response in June 2019 (Fig. 1).

**Fig. 2:**
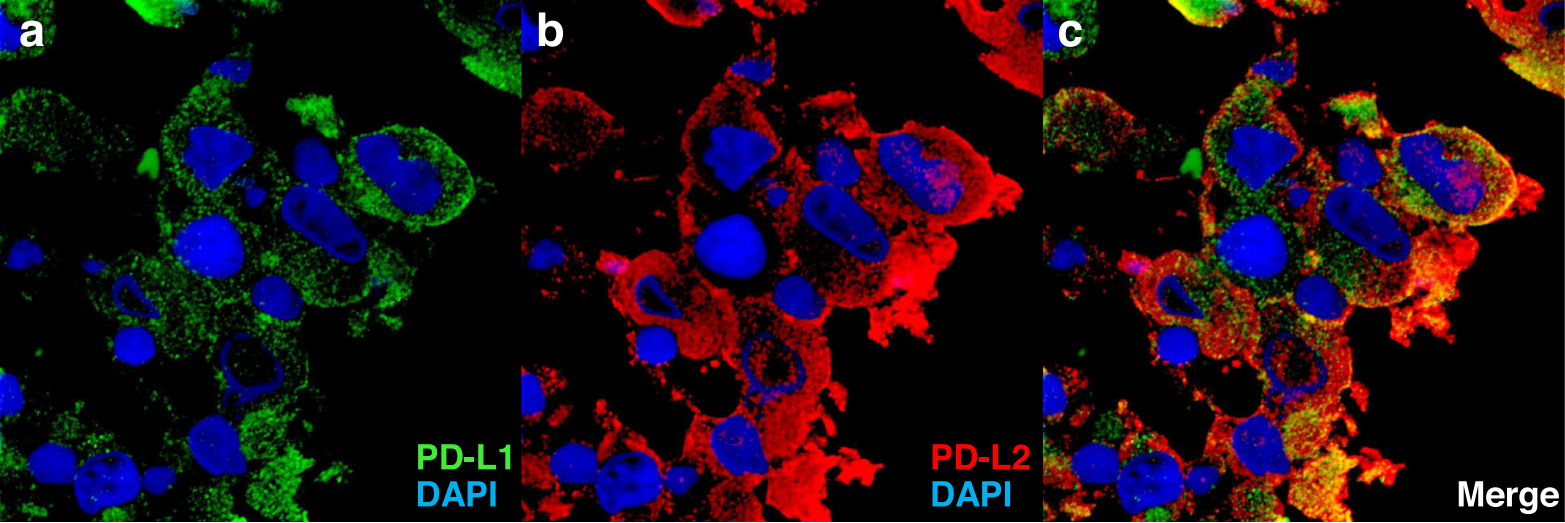
Immunofluorescence analysis of programmed cell death ligand 1 (PD-L1) and PD-L2 expression. The panels show fluorescence captions of PD-L1 (**a**, green) and PD-L2 (**b**, red) positivity of the same sample (**c**). Nuclei were stained with 4′, 6-diamidino-2-phenylindole (blue)

We analyzed the PD-L2 expression, and immunofluorescence double-staining showed high expression of PD-L1 and PD-L2 in the tumor tissue (Fig. 2, Supplementary methods of Fig. 2).

## Discussion and conclusions

The case presented herein adds valuable insights into the use of pembrolizumab in the setting of disease progression during durvalumab consolidation therapy after chemoradiotherapy in a patient with stage III SC with high PD-L1 expression.

The primary site in the right lung was reduced markedly, but three new metastatic lesions appeared during durvalumab treatment (Fig. 1). This case was clearly considered a progressive disease. The results of PACIFIC phase III trial are expected to establish durvalumab, a selective PD-L1 inhibitor, as the standard consolidation strategy in patients with unresectable stage III NSCLC without disease progression after concurrent chemoradiotherapy [8, 9]. Exploratory post-hoc analyzes showed that the benefits of PFS and OS in the durvalumab group were evident in patients with a PD-L1 tumor proportion score of 25% or greater before chemoradiation. PD-L1 expression of ≥25% on tumor cells occurred in 24.2% of patients in the durvalumab group, but the proportion of PD-L1 expression in ≥50% of tumor cells is unknown. Currently, as a result of the KEYNOTE-024 trial results, pembrolizumab, a selective PD-1 inhibitor, is administered as first-line treatment for patients with advanced NSCLC with PD-L1 expression on ≥50% of tumor cells [10]. One of the reasons that the efficacy of pembrolizumab is improved compared to durvalumab is the essential difference between anti-PD-1 and anti-PD-L1. Duan J. et al. performed meta-analysis and suggests that anti–PD-1 exhibited favorable survival outcomes and a safety profile comparable to that of anti–PD-L1 [11]. Pembrolizumab binds PD-1 and blocks the PD-1–PD-L1/PD-L2 axis. In contrast, durvalumab selectively blocks PD-L1 binding to PD-1 and CD80 without inhibiting PD-L2. Therefore, the interaction of PD-1 and PD-L2 remain intact and may inhibit T cell activation. In a recent report of patients with NSCLC treated with anti-PD-1 antibodies, both PD-L1 and PD-L2 positivity potentially predicted clinical response to anti-PD-1 therapy [12].

In the PACIFIC trial, 52.9% of the patients in the durvalumab group had a nonsquamous histologic type of tumor, but no cases of SC were reported. SC shows highly aggressive behavior and resistance to chemotherapy [3, 4]. Recent studies report that SC shows high levels of PD-L1 [5], tumor mutation burden and strong immune-cell infiltration [6]. We suspected pleomorphic carcinoma in this case because of the histologic components of spindle and giant cells, although there was limited biopsy tissue. Pulmonary pleomorphic carcinomas very frequently express both PD-L1 and PD-L2 [13]. This case had high PD-L1/PD-L2 expression on tumor cells (Fig. 2). Therefore, targeting the PD-1–PD-L1/PD-L2 pathway may represent a potential therapeutic candidate for this type of aggressive tumor after failure of durvalumab. In conclusion, pembrolizumab may be an option for treatment to durvalumab resistance after definitive chemoradiotherapy in a patient with stage III SC with high PD-L1 and PD-L2 expression. Of course, this case is only one case, so we cannot assert it and more cases are needed.

## Supplementary information

**Additional file 1.** Supplementary methods of Fig. 2 The methods of Immunofluorescence analysis of PD-L1 and PD-L2 expression.

## Data Availability

All data are contained within the manuscript.

## Abbreviations

SC: Sarcomatoid carcinoma
NSCLC: Non-small-cell lung cancer
PD-L1: Programmed cell death ligand 1
PD-L2: Programmed cell death ligand 2
CT: Computed tomography
PFS: Progression-free survival
OS: Overall survival
