# Supplementary material for: Favorable response to pembrolizumab after durvalumab failure in a stage III sarcomatoid carcinoma of the lung: a case report
Source: BMC Pharmacol Toxicol. 2020 Apr 3;21:26. doi: 10.1186/s40360-020-00404-7 (PMC7118808; doi:10.1186/s40360-020-00404-7)
Supplement: Supplementary file 1 — Additional file 1. Supplementary methods of Fig. 2 The methods of Immunofluorescence analysis of PD-L1 and PD-L2 expression. [file 40360_2020_404_MOESM1_ESM.docx]

**Supplementary Methods of Figure 2**

We prepared tissue specimens of 3-μm thickness from formalin-fixed paraffin-embedded biopsy samples. The specimens were deparaffinized followed by heat-induced epitope retrieval, washed in cool running water and Tris-buffered saline (pH 7.4), and incubated with the relevant primary antibodies (Abs) according to the manufacturers’ protocols. Monoclonal Abs specific for PD-L1 (#ab210931, abcam, Cambridge, UK) and PD-L2 (#MAB1224, R&D, Minneapolis, MN, USA) were used. Then the slides were incubated with appropriate secondary Abs: Alexa Fluor 488-conjugated anti-rabbit and Alexa-Fluor 647-conjugated anti-mouse (Thermo Fisher Scientific Inc, St Louis, MO, USA). After subsequent washing, the sections were mounted with Prolong Gold antifade reagent with 4’,6-diamidino-2-phenylidole (Thermo Fisher Scientific Inc) and coverslips were applied. Appropriate isotype-matched controls were prepared.
